# Resilient care in times of covid: The stress buddy

**DOI:** 10.1192/j.eurpsy.2021.836

**Published:** 2021-08-13

**Authors:** N. Rius-Ottenheim, E. Vermetten, E. Giltay, M. Boeschoten, N. De Bles, N. Van Der Wee, A. Van Hemert

**Affiliations:** 1 Psychiatry, LUMC, Leiden, Netherlands; 2 Psychotrauma, Arq Foundation, Diemen, Netherlands

## Abstract

**Introduction:**

The COVID-19 outbreak poses a challenge for health care professionals due to a surge in care demands, overwork, fear of contagion and concerns on the availability of protective equipment, and coping with distress of patients and their families. Although there is emerging evidence on prevalence of stress and its predictors, less is known on the trajectory of stress symptoms and the differences between cohorts of health care professionals.

**Objectives:**

To sustain and restore health care professionals the Leiden University Medical Center has launched the Digital Stress Buddy, a mobile app, to assess psychological stress, depressive symptoms, anxiety and posttraumatic stress symptoms.

**Methods:**

Participants fill in a 14-item questionnaire on stress and resilience resources, followed by a COVID-related questionnaire and finally a set of validated questionnaires on depression and anxiety (DASS-21), posttraumatic stress-symptoms (IES-R), burn-out (CBI) and resilience (RES).

**Results:**

To date, 959 health care workers have completed the stress monitor, of whom 223 (23%) showed relevant stress levels. Within this group, anxiety and posttraumatic symptoms were most prevalent (45%), followed by depressive symptoms (15%). Predictors of stress were being female, coping with distress of patients and their families, teleworking, and overwork.

**Conclusions:**

By identifying vulnerabilities and resilience for psychological distress, we are able to tailor the support interventions for health care workers within our hospital. This is an ongoing study and future follow-up during the second wave of the pandemic will provide more insight on the trajectories of stress-related symptoms.

**Conflict of interest:**

No significant relationships.

